# First imported case of *Candida auris* infection in Milan, Italy: genomic characterisation

**DOI:** 10.1007/s15010-024-02232-x

**Published:** 2024-04-01

**Authors:** Sara Giordana Rimoldi, Riccardo Nodari, Alberto Rizzo, Alessandro Tamoni, Concetta Longobardi, Cristina Pagani, Silvia Grosso, Federica Salari, Laura Galimberti, Pietro Olivieri, Giuliano Rizzardini, Emanuele Catena, Spinello Antinori, Francesco Comandatore, Antonio Castelli, Maria Rita Gismondo

**Affiliations:** 1 Clinical Microbiology, Virology and Bioemergencies, ASST Fatebenefratelli Sacco, Milan, Italy; 2 Department of Biomedical and Clinical Sciences, Romeo ed Enrica Invernizzi Paediatric Research Centre, University of Milan, Milan, Italy; 3 Dipartimento di Scienze Biomediche e Cliniche, ASST Fatebenefratelli Sacco, Università di Milano, Via Giovanni Battista Grassi n° 74, 20157 Milan, Italy; 4 III Division of Infectious Diseases, ASST Fatebenefratelli Sacco, Milan, Italy; 5 Medical Direction Unit, ASST Fatebenefratelli Sacco, Milan, Italy; 6 Department of Infectious Diseases, ASST Fatebenefratelli Sacco, Milan, Italy; 7 Anestesiology Unit, ASST Fatebenefratelli Sacco, Milan, Italy

**Keywords:** *Candida auris*, Yeast, Multi-Drug-Resistance-Organism, Whole-Genome-Sequencing, Italy

## Abstract

**Purpose:**

*Candida auris*, an emerging multidrug-resistant yeast, has been reported worldwide. In Italy, the first case was reported in 2019. We describe the first case of *C. auris*, imported from Greece, in Milan, using whole genome sequencing to characterise mutations associated with antifungal resistance.

**Case presentation:**

On October 2022 an 80-year-old Italian man was hospitalised in Greece. In the absence of clinical improvement, the patient was transferred to our hospital, in Italy, where blood culture resulted positive for *C. auris*. Despite therapy, the patient died of septic shock. In a phylogenetic analysis the genome was assigned to Clade I with strains from Kenya, United Arab Emirates and India. D1/D2 region resulted identical to a Greek strain, as for many other strains from different World regions, highlighting the diffusion of this strain.

**Conclusion:**

Importation of *C. auris* from abroad has been previously described. We report the first case of *C. auris* imported into Italy from Greece, according to phylogenetic analysis. This case reinforces the need for monitoring critically ill hospitalised patients also for fungi and addresses the need for the standardisation of susceptibility testing and strategies for diagnosis and therapy.

*Candida auris* is an emerging multidrug-resistant yeast that presents a high rate of treatment failure. *C. auris* often exhibits resistance to fluconazole, but variable susceptibility to other azoles, such as voriconazole, itraconazole or amphotericin B, and echinocandins have been observed [1]. Since its emergence, *C. auris* cases have been reported all over the world: from 2013 to 2021, 1812 clinical cases were reported by 15 EU/EEA countries while 3270 infection cases and 7413 screening-positive cases were documented in the USA through 31 December 2021 [2, 3]. In Italy the first case was reported in 2019, followed by sporadic cases and a major outbreak in healthcare facilities of the Liguria region, northern Italy, in 2021 [2, 4].

We describe the first case of *C. auris* imported from Greece in Milan (Lombardy, Italy) through the use of whole genome sequencing (WGS) to characterise mutations associated with antifungal resistance.

In October 2022 an Italian 80 years old man affected by diabetes and hypertension received a diagnosis of West Nile Virus encephalitis in Greece and required 5 months hospitalisation in the Intensive Care Unit (ICU). On May 2023 blood culture turned positive for NDM-producing *Klebsiella pneumoniae* and *C. auris*; the latter grew also from skin swabs collected for surveillance.

On 16 May 2023 the patient was transferred to our ICU in Italy for respiratory failure. At the time of admission colonisation on multiple sites (skin, rectum) by multidrug-resistant microorganisms, including *K. pneumoniae* NDM and *Acynetobacter baumannni* carbapenemase-resistant. *C. auris* was isolated from blood, tracheostomy tube and catheter tip culture. All measures of infection control were put in place: active surveillance for ICU rooms surfaces and ICU patients was adopted. During the short hospital stay in our ICU blood culture became positive for *C. auris* and *K. pneumoniae (*blaNDM gene) but despite prompt antimicrobial therapy (including caspofungin treatment) the patient died of septic shock two days later.

*C. auris* strain was isolated from blood culture (BioMérieux, France) after culture on Sabouraud Dextrose agar (Becton Dickinson, USA) for 48 h at 30 °C.

*C. auris* species identification has been carried out by MALDI-TOF device on Vitek MS (BioMérieux). Antifungal susceptibility testing of amphotericin B, flucytosine, fluconazole, itraconazole, voriconazole, posaconazole, caspofungin, anidulafungin, micafungin was performed according to EUCAST [5]. Sequencing was performed through the Ion Xpress Plus gDNA Fragment Library Kit (Thermo Fisher) on the Ion GeneStudio™ S5 System (Life Technologies, USA). The obtained reads were assembled using Spades v3.9.0. *C. auris* genome was submitted to the NCBI database (MIL-ITA-2023).

The protein sequences of the resistance genes were retrieved from the *C. auris* B8441 NCBI genome assembly (PEKT02), used as a reference. The evaluation of resistance protein sequences was performed using tblastn. Genetic signature confirmed the antifungal phenotype as reported in Table 1, revealing a resistant profile against several azoles and flucytosine. Tentative MIC breakpoints from the USA Centers for Disease Control and Prevention were used for the interpretation [6].

**Table 1:** *Candida auris* phenotypic and genotypic data about antifungal drugs obtained through susceptibility testing and whole genome sequencing (WGS)

Antifungal susceptibility test			WGS
Drug	MIC values isolate	Tentative MIC breakpoints	SNPs drug resistance
Amphotericin B	0.5	≥ 2	
Caspofungin	0.125	≥ 2	
Micafungin	0.06	≥ 4	
Voriconazole	0.06	N/A	
Anidulafungin	0.03	≥ 4	
Flucytosine	32		S70R (FCY1)
Fluconazole	> 32	≥ 32	Y132F (ERG11)
Itraconazole	> 4		M220L (ERG11)
Posaconazole	≥ 8		G54W (ERG11)
			I440V (ERG11)
			T55A (DHPS)

Column 1 shows antifungals tested in vitro against *C. auris* strain. Column 2 shows the Minimum Inhibitory Concentration (MIC) values (ug/mL) of the isolate. Column 3 contains tentative MIC breakpoints (https://www.cdc.gov/fungal/candida-auris/c-auris-antifungal.html, accessed on 23 Jan 2024). In column 4 are summarized the Single Nucleotide Polymorphisms (SNPs) of genes involved in antifungal resistance.

*S* susceptible, *R* resistant

Protein sequences of the resistance genes investigated: *DHFR* (PIS58359.1), *TUB2* (PIS51625.1), *FKS1* (PIS58465.1), *CYP51b* (PIS56902.1), *FKS2* (PIS58465.1), *ERG11* (PIS56902.1), *CYP51* (PIS56902.1), *DHPS* (PIS56787.1), *MfCYP51* (PIS56902.1), *CYP51c* (PIS56902.1), *CYP51a* (PIS56902.1), *COX10* (PIS49681.1), *RTA2* (PIS58632.1), *FUR1* (PIS52459.1), *ADE17* (PIS55745.1), *FCY1* (PIS48695.1) and *FCY2* (PIS55633.1)

Flucytosine resistance has been associated with mutations on *ADE17* + *FUR1* and *CrcB* + *FCY2* genes: the strain, however, expressed flucytosine resistance harbouring a single mutation in the *FCY1* gene; azoles resistance was consistent with mutations detected on gene *ERG11* [2]. *C. auris* did not exhibit intrinsic resistance to amphotericin B and echinocandins. Our strain showed minimum inhibitory concentration (MIC) values within the ranges observed in the 2019–-2022 Italian isolates for five out of nine antifungal drugs tested. MIC values of MIL-ITA-2023 showed different range for flucytosine, posaconazole, voriconazole and itraconazole [7]. To better investigate the origin of the MIL-ITA-2023 isolate a phylogenetic analysis was performed on a total number of 133 genomes from 15 countries on five continents.

The MIL-ITA-2023 isolate was then assigned to *Candida auris* Clade I and it is placed basal to a highly supported monophylum that includes strains from Kenya, United Arab Emirates and India (Fig. 1).

**Fig. 1:**
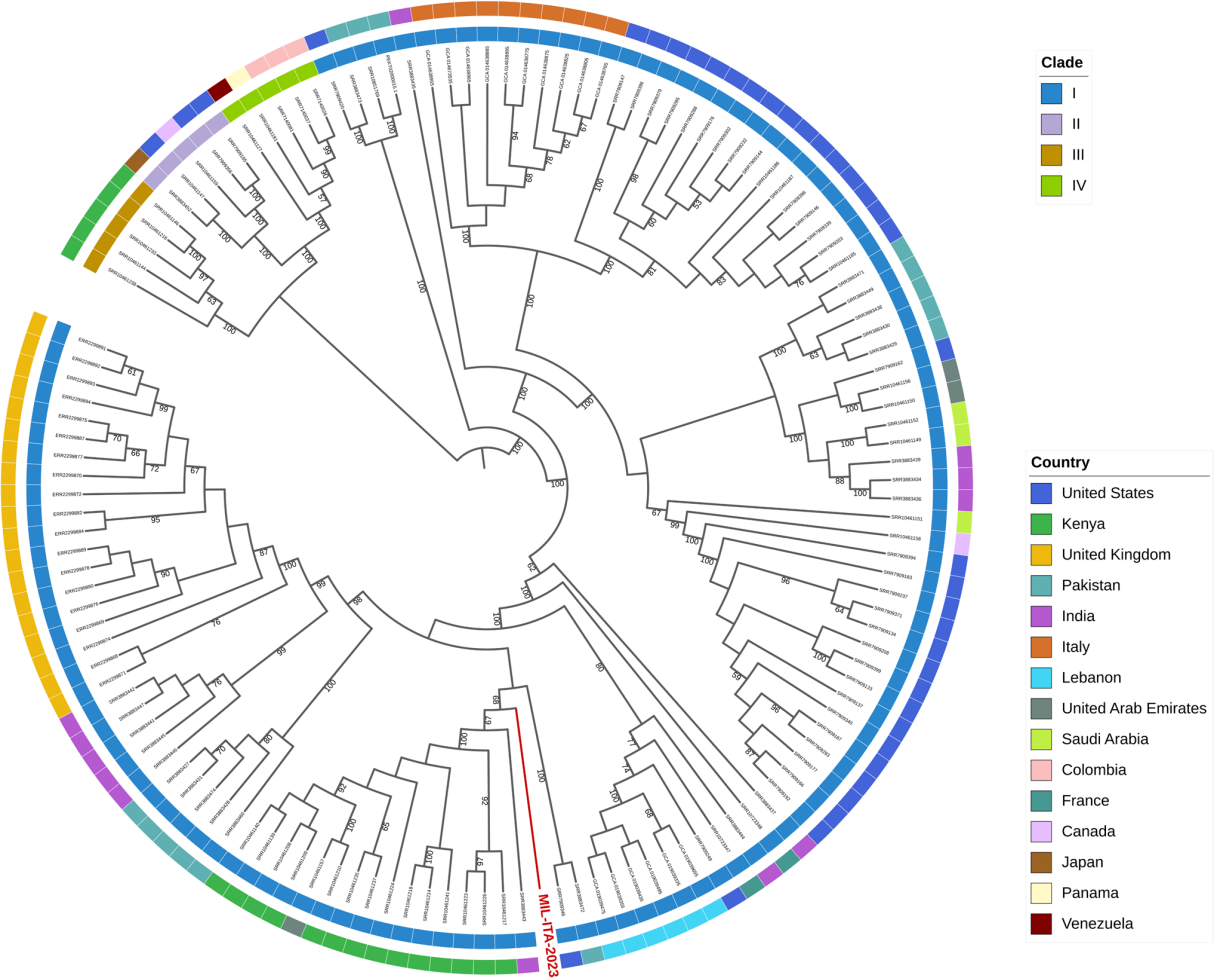
Four-clades population structure of *C. auris* using 133 genomes are represented in the Phylogenetic tree. Numbers above branches are bootstrap values (only values > 50 are shown)

The genetic distance between these genomes and the MIL-ITA-2023 genome was computed using the Mash tool and our strain was attributed to the genetically nearest clade. Blast was used to find in the NCBI nt database the genomes that were more similar to the study genome, which were then added to the background genomic dataset after manual curation. The analysis included (i) 117 published genomes [8], (ii) six genomes genetically close to the study strain, retrieved from NCBI after blastn search, (iii) 10 *C. auris* genomes isolated in Italy [9]. SNP calling was carried out using the Purple tool [10] and phylogenetic analysis was performed using RAxML8 [11] using the TVM evolutionary model, previously determined using ModelTest-NG [12]. The phylogenetic analysis indicated that MIL-ITA-2023 did not cluster with available *C. auris* genomes from Italy, consistent with the hypothesis that the patient was colonised outside our country.

Recently, a *C. auris* strain isolated in Greece has been phylogenetically characterised on the basis of the D1/D2 large subunit (LSU) rDNA and internal transcribed spacer (ITS) sequences [13]. Thus, we performed phylogenetic analyses on both the D1/D2 large subunit (LSU) rDNA-derived sequence and the ITS sequence of *C. auris*. The D1/D2 region sequence of strain MIL-ITA-2023 resulted in identical to the Greek strain (GenBank accession number MK975461) [13], as for many other strains from different world regions (Fig. 2). Similarly, the analysis of the ITS region revealed a striking resemblance between the sequence of strain MIL-ITA-2023 and the Greek strain (GenBank accession number MK981227) [9], as well as numerous other strains across diverse global regions (Fig. 3). Considering the patient's recent whereabouts, it would be useful to compare MIL-ITA-2023 genome with the Greek ones. Unfortunately, the absence of publicly available *C. auris* genomes isolated in Greece makes this comparison currently impossible to perform.

**Fig. 2:**
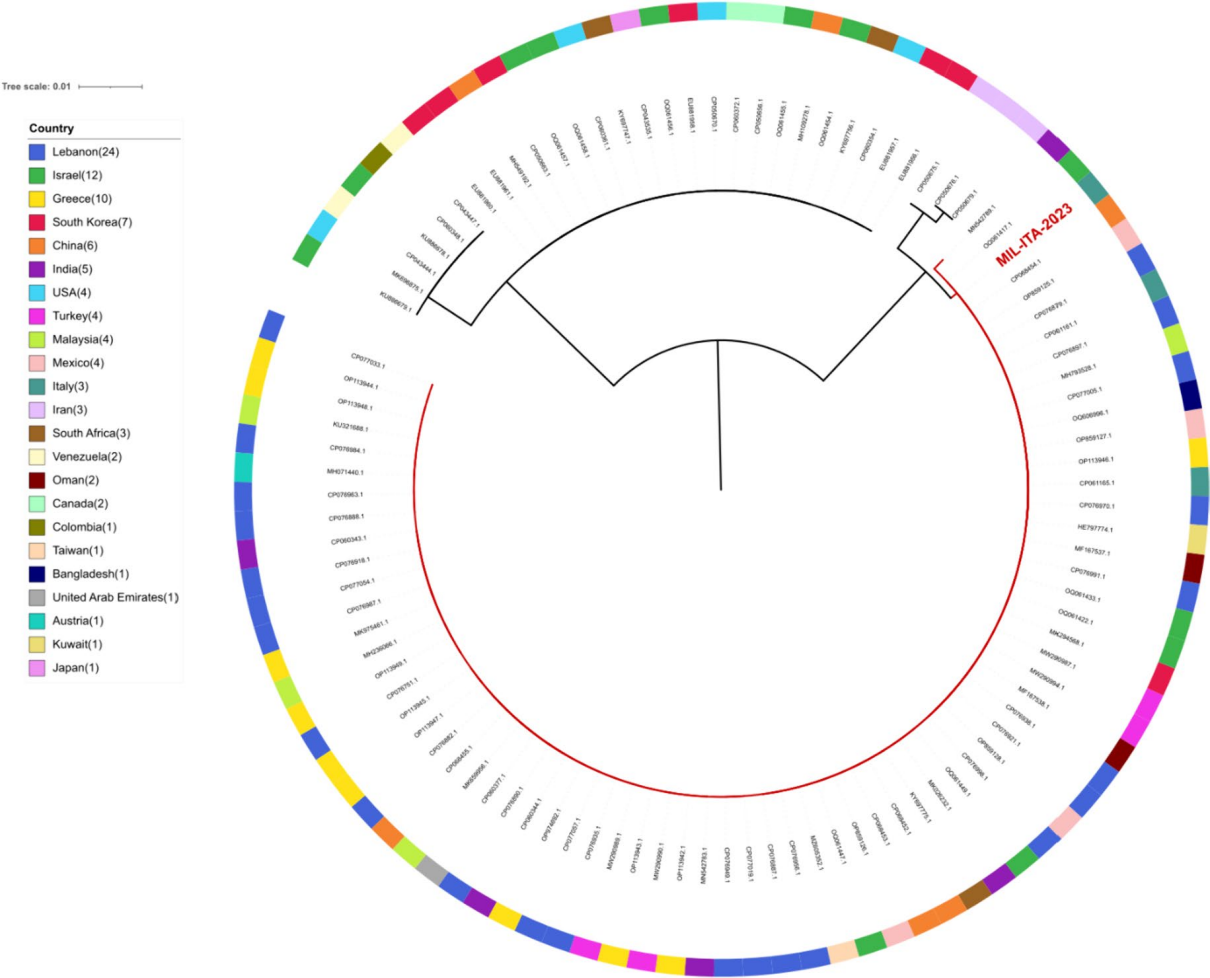
Maximum-likelihood phylogenetic tree on the partial D1/D2 large subunit (LSU) rDNA sequence of *Candida auris.* Sequences most similar to the D1/D2 rDNA sequence from Stathi et al. (2019) (GenBank accession number MK975461) were retrieved through blast analysis and analysed to determine the relation with the MIL-ITA-2023 sample (in red)

**Fig. 3:**
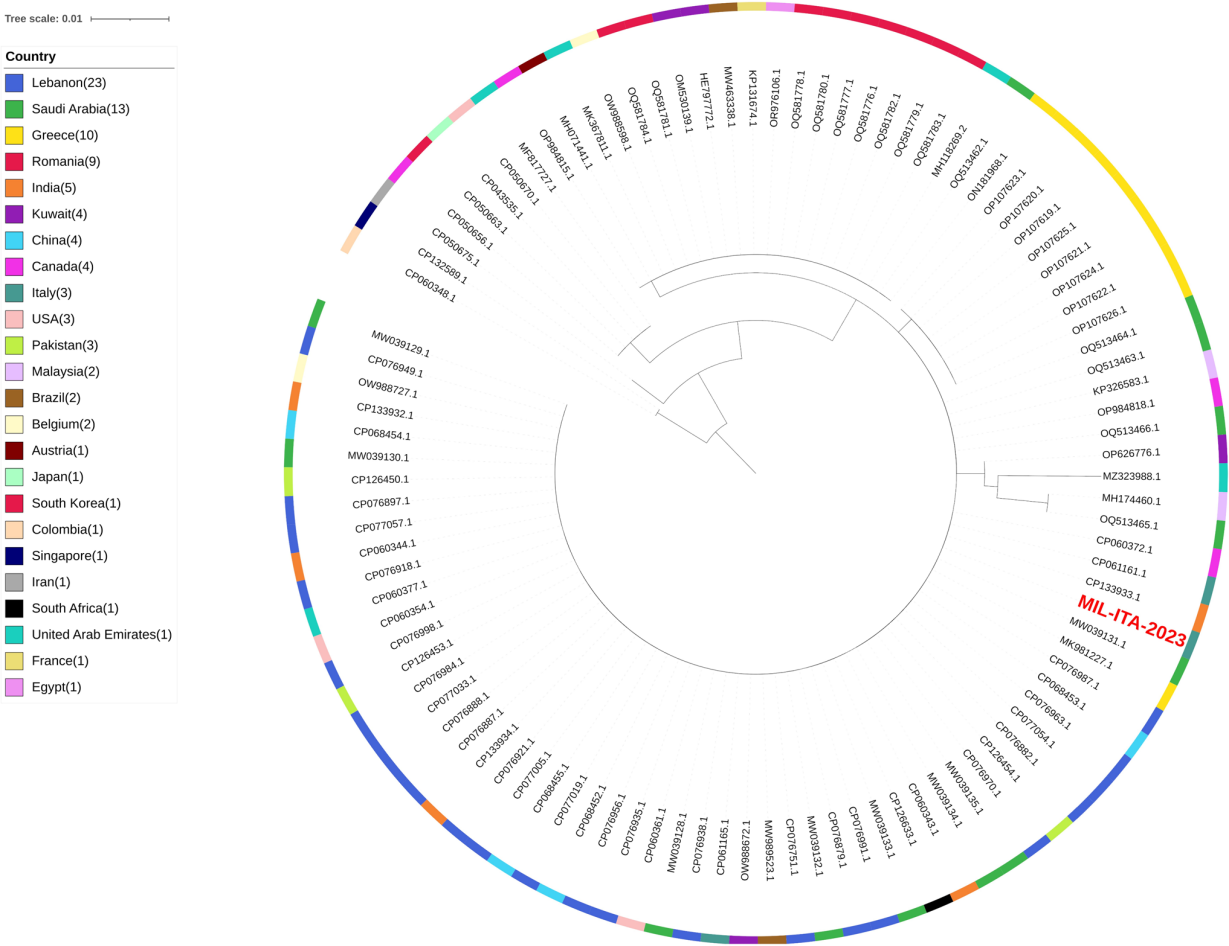
Maximum-likelihood phylogenetic tree on the internal transcribed spacer (ITS) sequence of *Candida auris*. Sequences most similar to the ITS sequence from Stathi et al. (2019) (GenBank accession number MK981227) were retrieved through blast analysis and analysed to determine the relation with the MIL-ITA-2023 sample (in red)

The result confirms the highly diffusion of this strain around the globe. Importation of *C. auris* has been previously described [14, 15]. All these cases share a prolonged hospitalisation in countries with a high rate of multidrug-resistant organisms (MDROs), the need for mechanical ventilation and the use of central venous catheters which can be colonised by *C. auris*. It should be highlighted that such cases can be the initial chain responsible for subsequent local transmission and therefore *had hoc* screening should be put in place as recently indicated by the Italian Ministry of Health. However, only one out of 351 cases detected in Italy in four regions (Liguria, Piedmont, Emilia-Romagna and Veneto) between July 2019 and December 2022 had a history of travel abroad. Interestingly, our strain maintained susceptibility to echinocandins despite having received several cycles of echinocandins therapy during the ICU stay in Greece.

The in-hospital mortality of *C. auris* was reported to be 40.3% in Italy and it does not differ from that described for other *Candida* species [7, 16]. *C. auris* represents a significant threat in healthcare settings. Due to high mortality and transmissibility and the multi-drug resistant profile of the microorganism, early identification of *C. auris* is crucial for timely treatment, along with the implementation of infection prevention and control measures. Some limitations in *C. auris* early identification include misidentification with other *Candida* species, laboratory capacities and technical skills. Another point that deserves to be considered about *C. auris* other than the challenge of correct and rapid identification is the fact that 1,3-β -D-glucan, important for the detection of candidemia among critically ill patients, shows a lower sensitivity in identifying bloodstream invasive infection caused by this yeast [17]. All laboratories might benefit from the introduction of reliable, specific and robust polymerase chain reaction assays that, however, are under evaluation [18, 19].

In conclusion, the combination of epidemiological and phylogenetic data strongly supports the hypothesis that the strain was imported from Greece. This is also the first genomic characterisation of *C. auris* case in the Lombardy region, despite the recent epidemics of *C. auris* infection that ravaged other regions of Northern Italy between 2019 and 2022.

The rapid implementation of infection control measures spared up to now the possible dissemination of such pathogen in our ICU. The increasing spread of *C. auris* in Italy starting in 2019 highlights the importance of local and international antifungal surveillance protocols. In Italy, the implementation of a national surveillance system that require reports for confirmed cases of colonisation or infection might represent a valuable system for *C. auris* epidemiology [20]. This case reinforces the need for monitoring ICU patient’s hospitalization and also for fungi and addresses the need for the standardization of susceptibility testing interpretation and strategies for diagnosis and therapy.

## Data Availability

Anonymised data used to perform the analysis will be provided upon (reasonable) request.
